# From the factory to the field: considerations of product characteristics for insecticide-treated net (ITN) bioefficacy testing

**DOI:** 10.1186/s12936-021-03897-7

**Published:** 2021-09-06

**Authors:** Ole Skovmand, Duoc M. Dang, Trung Q. Tran, Rune Bossellman, Sarah J. Moore

**Affiliations:** 1 Intelligent Insect Control, Castelnau le Lez, France; 2 Biolytrics Laboratories, Hanoi, Vietnam; 3 Vegro Aps, Copenhagen, Denmark; 4 Vector Control Product Testing Unit, Ifakara Health Institute, P.O. Box 74, Bagamoyo, Tanzania; 5 Department of Epidemiology and Public Health, Vector Biology Unit, Swiss Tropical and Public Health Institute, Socinstrasse, 57, 4002 Basel, Switzerland; 6 University of Basel, Petersplatz 1, 4001 Basel, Switzerland

**Keywords:** Insecticide-treated bed net, Long-lasting insecticidal nets, LLIN, Test methods, ITN specifications, Bioefficacy, Wash resistance index, WRI

## Abstract

**Background:**

Insecticide-treated nets (ITNs) undergo a series of tests to obtain listing by World Health Organization (WHO) Prequalification. These tests characterize the bioefficacy, physical and chemical properties of the ITN. ITN procurers assume that product specifications relate to product performance. Here, ITN test methods and their underlying assumptions are discussed from the perspective of the ITN manufacturing process and product characteristics.

**Methods:**

Data were extracted from WHO Pesticide Evaluation Scheme (WHOPES) meeting reports from 2003 to 2017, supplemented with additional chemical analysis to critically evaluate ITNs bioassays with a focus on sampling, washing and wash resistance, and bioefficacy testing. Production methods for ITNs and their impact on testing outcomes are described.

**Results and recommendations:**

ITNs are not homogenous products. They vary within panels and between the sides and the roof. Running tests of wash resistance using a before/after tests on the same sample or band within a net reduces test variability. As mosquitoes frequently interact with ITN roofs, additional sampling of the roof when evaluating ITNs is advisable because in nets where roof and sides are of the same material, the contribution of roof sample (20–25%) to the average is less than the tolerance for the specification (25%). Mosquito mortality data cannot be reliably used to evaluate net surface concentration to determine regeneration time (RT) and resistance to washing as nets may regenerate beyond the insecticide concentrations needed to kill 100% of susceptible mosquitoes. Chemical assays to quantify surface concentration are needed. The Wash Resistance Index (WRI) averaged over the first four washes is only informative if the product has a log linear loss rate of insecticide. Using a WRI that excludes the first wash off gives more reliable results. Storage conditions used for product specifications are lower than those encountered under product shipping and storage that may exceed 50 °C, and should be reconsidered. Operational monitoring of new ITNs and linking observed product performance, such as bioefficacy after 2 or 3 years of use, with product characteristics, such as WRI, will aid the development of more robust test methods and product specifications for new products coming to market.

## Background

Public health interventions with an existing World Health Organization (WHO) recommendation, including pyrethroid insecticide-treated nets (ITNs), undergo a WHO prequalification (PQ) process (previously WHO Pesticide Evaluation Scheme, WHOPES) to ensure their performance and quality. ITNs must provide a barrier against mosquito bites and also continue to kill mosquitoes even after they have been repeatedly washed by users, up to 20 times. WHO PQ listing is relied upon as a benchmark by donor funded procurement agencies such as Global Fund [1] and is, therefore, a critical step in getting ITN products to market for use in malaria vector control. To gain PQ listing, manufacturers generate a dossier of data from a series of bioefficacy performance tests that include laboratory studies and small scale field trials based on WHOPES guidelines [2].

The WHOPES guidelines for evaluating long-lasting insecticidal nets (LLIN) were developed in 1999–2000 in a co-operation between the Montpellier WHO reference laboratory at the Institute of Research and Development (IRD) at the Laboratories des Insectes Nuisibles (LIN) and the WHO reference chemical laboratory in Gembloux, Belgium. Their aim was to (1) provide specific and standardized procedures for testing ITNs for malaria vector control, and (2) harmonize the testing procedures carried out to ensure conformity of data used for registration and labelling of ITNs [3]. These were then updated in 2013 [2] to include new developments in measuring ITN performance. Since then new product classes have become available [4], and new procedures to evaluate them have been developed by many scientists in the product testing sphere and harmonized by entities, such as President’s Malaria Initiative and Innovation to Impact, in the absence of formal guidance. For pyrethroid ITNs, the 2013 guidelines [2] are followed.

The 2013 guidelines divide ITN evaluation into 3 phases. The first included evaluation of manufacturer-produced data on toxicology and physical parameters, with additional chemical and bioefficacy evaluation overseen by WHOPES in recognized laboratories. The results were compared to the specifications of the product and minimum performance criteria defined by WHOPES. The second phase was a semi field (experimental hut) test against wild populations of target vector mosquitoes where some of the results of phase one were used to define test parameters. Upon meeting bioefficacy criteria (equivalent or better performance than a WHOPES-recommended net) in WHOPES supervised studies, an interim recommendation was given. In order to receive full recommendation as a LLIN, the ITN underwent the third phase of testing. This consisted of large prospective longitudinal field studies of ITN performance under user conditions.

Thresholds for bioefficacy were based on early trials of Olyset^®^ LLINs [5]. Observed public health value (i.e. reduction in malaria among communities that received ITNs) corresponded with entomological indices measured in (1) field trials (reduced vector density, sporozoite rate and parity) [5]; (2) experimental hut trials (high mortality and feeding inhibition exceeding that on dipped nets) [6, 7]; and (3) laboratory bioassays (> 80% knock down, 70–80% mortality in cone test). The tunnel test was later introduced to evaluate blood feeding inhibition in the laboratory, and was shown to agree with data using lab mosquitoes released in experimental huts [8]. Careful studies were conducted to directly compare pyrethroids used on ITNs in cone and tunnel tests and better understand their mode of action (mosquito mortality, knock-down effect, irritancy, and blood-feeding inhibition) [9]. The longevity of ITNs was tested by washing them 20 times based on an estimated lifespan of 3 years (assuming nets are washed an average of once every few months). This resulted in a simple and robust testing modality by which ITN efficacy is verified, and products with the same or similar active ingredients (AIs) can be compared.

At the time of the first guideline development, a long-lasting coating formulation for a polyester net (PermaNet) had been developed. The WHOPES guidelines were based primarily on this coating formulation and did not consider the basic differences of another method of making ITNs: the incorporation technology that was already used in Olyset. This older product was not formally evaluated according to the guidelines [5], that were developed later in 2005 [3]. Chemical assays were used for net specifications and to support the bioassays.

There are now 22 prequalified ITNs made from a number of different materials, single AIs and also mixtures of AIs that may be incorporated or coated. The tests outlined in the WHO guidelines do not adequately consider the physical chemistry laws that govern migration of additives in coatings or from a polymer matrix, to the surface of the ITN where they are bioavailable. This article provides information on production technology that governs several aspects of ITN quality: yarn strength, batch variability, resistance to washing, resistance to high temperature is presented; and how sampling and test methods should consider the release/retention characteristics of the product to generate more consistent performance data is discussed.

### Methods for ITN production

The two main technologies used for producing ITNs are impregnation (coating) and incorporation of insecticide. Technologies used for incorporation and migration of any kind of additives are well described in the literature on food packing films. Coating technologies used in commercial nets are based on the mixing of insecticide particle suspensions in water with polymer suspension or a polymerizing suspension that then glues the insecticide particles to the yarns, while optionally adding a thin film covering these particles to slow AI loss through evaporation, wash-off and abrasion. The inherent physical strength of ITNs and their continued bioefficacy after exposure to washing, abrasion and high temperatures depends upon these production methods. The chemical laws for migration of insecticide from ITNs and the inherent product characteristics linked to their production technologies impact upon product performance, and should be considered when evaluating ITNs. It may not be appropriate to assume that all ITNs behave in the same way under the same test conditions. This is of particular importance when considering how insecticidal nets continue to perform after repeated washing that reduces surface concentration of insecticide.

### Production of polyester LLIN: impregnation

**Fig. 1:**
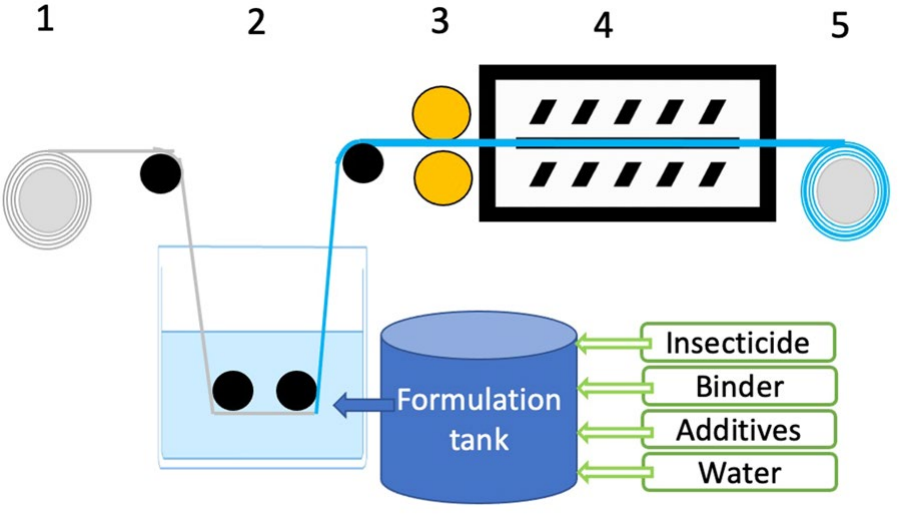
The foulard process for impregnated ITNs. The net starts on a roller (1) and is lead into a bath with insecticide suspension (2), then between two rollers pressed against each other (3) to squeeze out excess fluid. The net passes through a multisector oven where each section can be temperature regulated to heat cure it (4) and finally the finished net is rolled up again (5) ready for cut and sew

Polyester (Polyethylene Phthalate, PET) net technology are mostly based on coating the net in a foulard (bath) process followed by pressing off surplus suspension and drying the net in a long oven to evaporate the water and polymerize (cure) the oligomer suspension (Fig. 1). This results in an acryl or urethane based polymer coating affixing the insecticide onto the polyester filament. The technical term for this process is “impregnation”, though this word is sometimes (incorrectly) used for all ITNs.

**Fig. 2:**
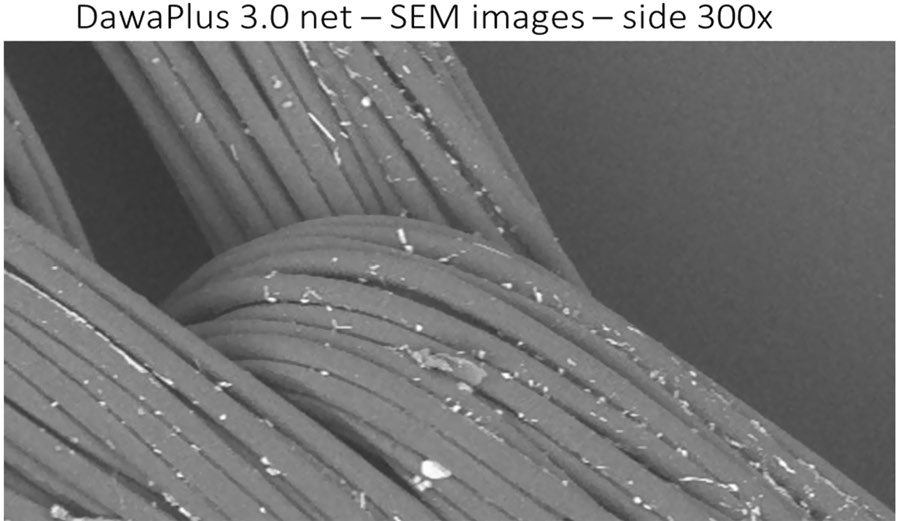
Scanning electron microscope image of a PET multifilament yarn coated with a dispersion of deltamethrin particles. The diameter of one filament is around 16 microns. It can be seen that the insecticide particles concentrate between filaments

Only deltamethrin and alpha-cypermethrin are used in impregnation of PET nets. These insecticides are crystalline at room temperature and the impregnation process consists of mixing a particle suspension received as a concentrate (suspension concentrate, SC) or producing SC using a technology called anti-solvent [10]. The SC is diluted with water then mixed with the detergents and the coating. When dried on the net, the insecticide particles will drift to the last wet point, which will be between the multifilament interface at the surface (Fig. 2). Release of the insecticide is from the crystal form to an amorphous form that migrates through the fine layer of coating and is exposed at the surface for insecticidal activity (and also available for wash off). The migration road is thus extremely short. Migration speed depends on the polymer or polymer mix used for the coating, the thickness of the coated layer, the insecticide, and the ambient temperature during storage and ITN use [11]. Since the curing process takes place at relatively high temperature, there will be more insecticide at the surface of a new net than ever after.

PET yarns consist of multiple filaments (typically 48 in 100 denier yarns) that are twisted together in the yarn production process. PET is a highly crystalline polymer. To be coloured, the colour is either added in the polymer before extrusion of the filaments, or colour is applied to the yarns or the knitted net at a later stage. To add colour after yarn extrusion, the PET yarn surface is treated with a caustic detergent solution that makes the surface of the yarn able to receive the colour particles, a method called reduction clearing [12]. This process also impacts the release of insecticide from the yarn [13], probably by irreversible catching of insecticide particles, and caused early concerns around the efficacy of coloured nets. However, white nets produced from the extrusion colour process do not have this issue, so they are often preferred by procurers (although they may not be preferred by users for numerous cultural reasons). The PET used for ITN manufacture exists in many varieties and qualities. Yarn strength can vary as measured in the tenacity test that measures the ability of a single yarn to resist a draw relative to the diameter of the yarn. Twisting of the yarn, number of filaments and brushing up the surface to create so-called texturized yarns all influence net bursting strength [14].

PET is hydrophobic, so to attach a water suspension to it, low surface tension detergents are used. Insecticide particles are heavier than water, so the foulard bath must be constantly stirred to ensure uniform AI dosage in the part of the bath where the net is passing. After that, the net is pressed between rollers to remove excess fluid and obtain target dosage of AI (Fig. 1). The textile rollers of the machines used for ITN production change elasticity as the rollers age. As a result, the press off at the sides can differ from that in the middle and if the rollers are not perfectly round, there will be small differences in press off during one rotation. This results in heterogeneity in AI content over the ITN. After passing between the rollers, the net goes into a long oven to be dried and to polymerize the coating principle (curing). The net is produced in widths that correspond either to the height of the bed net or the width of the roof. As these are often different, the roof and sides come from different productions and may be subject to batch variability. Sampling from PET nets for quality control must be adequate to reflect this potential for variability within nets due to the manufacturing process. It is also reflected in the relatively wide tolerance limit of the specification accepted for ITNs (± 25%).

### Production of polyethylene nets: incorporation

**Fig. 3:**
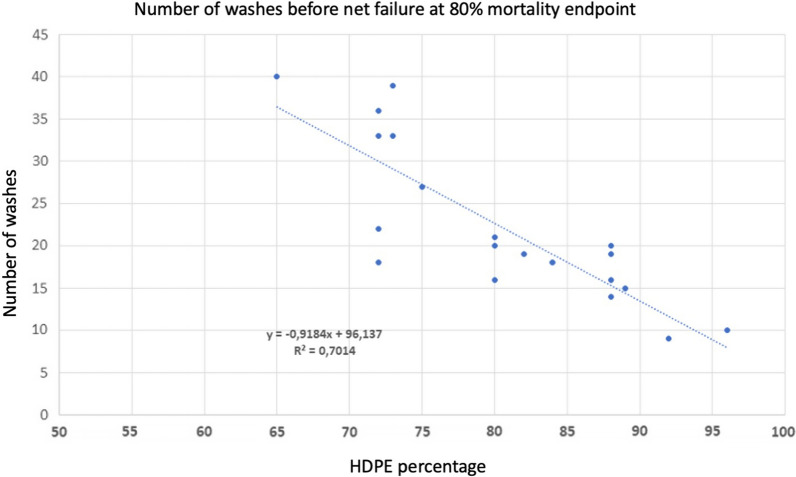
The ratio of LDPE and HDPE affects the release of insecticides from ITNs. The data show shows how many washes 20 experimental polyethylene (PE) nets could resist before failing according to WHO cone tests as a function of the High density PE (HDPE) percent of the polymer total, here using the 80% optimum mortality threshold. Besides High Density HDPE and low density LDPE and insecticide, the yarns contained up to 5 additives at various concentrations from 0 to 1%. The regression shows that 70% of the variation observed can be explained by the HDPE fraction or inverse, the LDPE fraction

Polyethylene (PE) nets are typically made from single filament yarns that are extruded with additives, colour, and AI. Therefore, the insecticide is located throughout the net yarn so the technology is called “incorporation”. PE yarns are typically made as a mix of high density and low density PE (HDPE and LDPE, respectively) or linear low density PE (LLDPE). These vary in structure: HDPE and LLDPE have short, linear side chains, whereas LDPE is branched and much less crystalline than the two others. HDPE is around 90% crystalline and LLDPE is 50–70% crystalline making it denser and stronger than LDPE. This difference in the level of branching within the polymer affects migration of AI through the fibres to the surface of incorporated nets where it becomes bioavailable to mosquitoes. The literature on migration of additives in PE is vast [11]. There are two theories for migration of additives in a polyethylene matrix. One considers a migration in the non-crystalline zones as a diffusion, the other anticipates that the insecticide in vapour form moves between holes between crystalline areas in polymer chains [15, 16]. The dense crystalline zones block the migration in both models, so HDPE has much lower migration rate of additives than LDPE. It, therefore, follows that the migration rate of an additive or an insecticide can be regulated by choosing a suitable ratio of LDPE and HDPE. This is demonstrated in Fig. 3 based on experimental data where 20 nets were made containing various ratios of HDPE and LDPE ranging from 65% HDPE: 35% LDPE to 95% HDPE:5% LDPE with a fixed dosage of insecticide per kg net. The nets were repeatedly washed and evaluated by cone bioassay with failure at the 80% mortality criterium. Figure 3 also shows that the net wash resistance is directly related to the ratio of LDPE and HDPE and if the net was heat cured or not with little effect from other additives.

Variations in polymer chain length in high- and low-density PE critically influence the migration of insecticide to the surface. This is considered in the melt index (MI) that is a measure for the viscosity at a certain temperature, and is used to optimize the relative mix of LDPE, LLDPE and HDPE in ITNs [17]. The higher the number, the lower the viscosity. However, MI does not fully explain insecticide migration rate in the polymer mix because the degree of branching also influences migration. Finally, the migration rate in the yarn is of course temperature dependent. PE is a thermoplastic, so the migration is temperature dependent in a non-linear way.

**Fig. 4:**
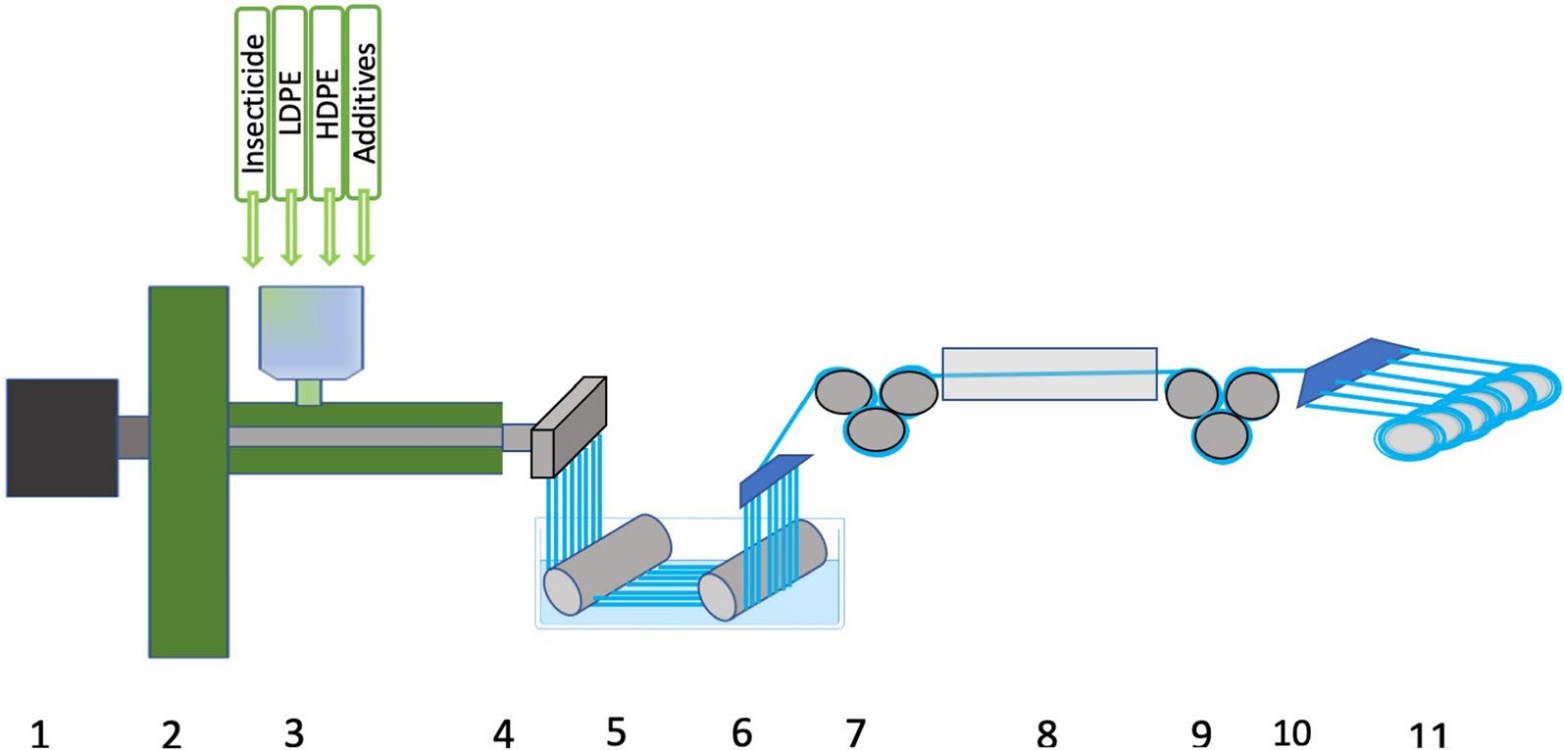
Production of incorporated ITNs. A simplified drawing of a round yarn polyethylene extruder. (1) the motor driving the extrusion screw. (2) the hopper containing mix of HDPE, LDPE and additives in Masterbatches, (3) the extruder tube with heating and eventually cooling bodies around a central tube with the extruder screw. (4) the die head where 150–300 yarns are extruded in parallel into a water bath (5). (6) a comb that arrange the yarns parallel drawn by the first set of rotating rollers (7). (8) a stretch zone where yarns are drawn by the difference in speed of rotation rollers (9) and (7). (10) a set of combs that guides the yarns to winding up on cones (11). Typically, there are 5–7 rotating rollers on each side of the bath and just as many cones as yarns produced at the die head. The hopper can be fed automatically from containers via a vacuum system or it can be premixed and hand-carried or pumped to the hopper. Extruder screws can have many designs to provide better mixing, but net producers mostly use very simple, single screws

Insecticide migration rate is also changed during stretching after extrusion (Fig. 4). Greater stretching of the yarn results in more highly packed and dense crystal zones of the yarn, resulting in higher yarn tenacity and lower migration rate [18]. As for PET, PE is found in many grades and these are not freely mixable. The wrong combination results in weak yarns and even yarn breakage during production. PE bed net yarns are typically made in extruders used for other technical yarns (including fishing net, agricultural nets, soil nets). Single screw extruders are used and the material is fed into a tray (“hopper”) at the start of the extruder, then heated in the extruder tube while being pressed forward to the “die head” where the mass is pressed through one or several “spinnerets” that are circles of small holes in solid iron plates (Fig. 4). Yarns are extruded from the polymer mix. The insecticide that is typically premixed as concentrate in pellets (masterbatches) is mixed into the polymer mix along with other additives in the same or additional masterbatches. However, single screw extruders are poor mixers. The standard procedure for obtaining a homogenous product from such extruders is to recycle the extruded product up to 5 times, but this is impossible for insecticide yarn production, since the insecticide is degraded at each passage and it would be prohibitively expensive for the ITN market. Following Reynolds law for movement of a fluid, the mass close to the sides of the extruder tube moves much slower than that in the centre. This can be further accentuated by the nature of the additives, e.g., the melting point of insecticides are well below the process temperature of the polymer, so these behave like low viscous oils in a high viscosity polymer mass. Consequently, yarn dosage varies over time, particularly in short production series that are performed during product development. During mass productions, variations are mostly a start-up problem that reduce over time. Therefore, yarns from the start should simply be discarded.

When the yarns are knitted to nets, yarns extruded at the same time typically end on the same bobbin that sits on one side of the knitting machine. These bobbins are 30–40 cm wide and thus provide a part of the yarns used for a stripe of net with approximately the same width. The other part comes from a bobbin sitting on the other side of the knitting machine, and the two sets of yarns are mixed in the knitting process. Therefore, a band of net corresponding to one set of 2 bobbins has small variation inside the 30–40 cm band, but can be quite different from a neighbour band where yarns have come from another extrusion time or even another extruder. When nets are sewn, the four sides typically come from one piece that includes 4 to 6 bands, whereas the roof comes from another piece that includes 3 to 5 bands. Relatively big differences can therefore be expected between the roof and the side samples. This should be reflected when sampling from PE nets for quality control. It is also reflected in the relatively wide (± 25%) tolerance limit of the specification accepted for ITNs.

### Considerations for ITN testing

The temperature at which products are evaluated in order to generate product specifications [19] are critical in ITN evaluation. There is considerable heterogeneity in the methods suggested. For example, insecticide formulation storage stability assessment (CIPAC 46.3 m) may be conducted at 54 ± 2 ºC for 14 days with alternative conditions: 4 weeks at 50 ± 2 ºC, 6 weeks at 45 ± 2 ºC; 8 weeks at 40 ± 2 ºC, 12 weeks at 35 ± 2 ºC or 18 weeks at 30 ± 2 ºC [19]. After elevated temperature storage the formulation must retain its properties including for ITNs its insecticide migration profile. For migration in simple systems these temperature/time settings may provide equivalent results but for thermoplastic polymer systems as used, i.e., incorporated and impregnated ITNs these equivalences are not valid. Nets may fail to meet requirements (retain 95% of AI) in storage tests at 54 °C but pass at 40 °C. The WHO/FAO advises that storage stability testing at the conditions recommended for storage is expected to provide a more reliable indicator. ITNs are often shipped by sea and storage containers range between 35ºC and may exceed 50 ºC [20].

When deltamethrin and alpha-cypermethrin insecticides (that are crystalline at room temperature) migrate from the yarn, they migrate in the molecular form, but recrystallize on the yarn surface. Therefore, at the yarn surface there is a mix of newly migrated insecticide in amorphous form and crystallized insecticide [21]. The insecticide in the crystal form cannot migrate back into the yarn so there will be an increased dosage at the surface over time, a process called “blooming”. The crystals formed at the surface can be seen in EM scanning (Fig. 2). This has important consequences for the efficacy of ITNs: the bioefficacy of the product mostly depends on the insecticide in molecular form [22]. In polyester, the migration from the cured crystal suspension will be influenced by the particle size. Smaller particles have bigger particle surface area to volume ratio, and faster migration rate. In polyethylene nets, the insecticide inside the yarn is in molecular form, and crystals are formed at the surface, but this is a loss, as the crystals have very little insecticidal effect [23]. Permethrin is an oil at room temperature so the release of permethrin can go to an equilibrium. Like the other pyrethroids, permethrin is very poorly dissolved in polyethylene, so the release process is extremely slow. When Sumitomo produced Olyset Plus, they changed the ratio of low and high density polymer in the yarn to improve the release rate of permethrin [17].

### Regeneration time methodology

For impregnated nets, all insecticide is present in a coating at the surface of the material, whereas in incorporated nets most of the insecticide is inside the polymer matrix. Upon removal by washing, new insecticide must migrate to the surface of the net to regain activity, i.e., regeneration. After removal of insecticide due to washing, the regeneration time becomes the parameter that determines how fast the ITN again becomes effective for mosquito control. WHOPES guidelines [2] outline a procedure to estimate ITN wash resistance over 20 washes based on this principle. The interval between washes is based on the regeneration time of the net. The test net is washed thoroughly to remove insecticide from the surface. The net is then bioassayed on seven successive days. The cone test is normally used (although tunnel test with longer holding times is used for pro-insecticides so that mosquitoes may be metabolically active [24, 25]). Cone test endpoints are percentage of Knock-Down after 1 h (KD60) and the percentage of mortality after 24 h. The time required (in days) to reach a stable mortality level is the period required for regeneration of the net. Nets are then washed 20 times using this wash interval and if the net is still meeting optimal bioefficacy criteria of ≥ 80% 24 h mortality or ≥ 95% KD60 after 20 washes at this regeneration time interval, it meets WHO quality standards. This interval is dependent on bioefficacy data since there are no reliable chemical tests that correspond to the surface concentration and bioefficacy of ITNs [26].

Reliance on bioefficacy to calculate the washing interval is problematic as seen from historical data in WHOPES reports. Time testing of Olyset with a one-day wash interval showed that the net lost insecticidal activity after 2 washes, but a heating for 1–4 h at 60 °C or waiting 15 days could re-establish optimal insecticidal activity [5, 27]. Conversely, PermaNet 1 met 50% mortality bioefficacy criteria after 20 washes with a one-day wash interval, but failed after 5 washes with 7-days interval [28]. PermaNet 1 had a regeneration time at 7 days or more [28]. Therefore, by increasing wash intervals from 1 to 7 days, not only more insecticide was exposed to mosquitoes in bioassays, but more was exposed to wash off. This issue with wash resistance lead to the development of PermaNet 2.0 that was wash resistant to 10 washes according to the mortality criterium, and 20 washes according to KD60 criterium even when washed with 7 days interval [28]. These results show that regeneration time is crucial to product performance in WHOPES bioassays, and that KD60 is observed at lower insecticidal concentrations than mortality. Indeed, it is reported that the KD60 criterium is met at a dosage 10 times lower than the mortality criterium [29]. Similarly, tunnel tests that are recommended for irritant insecticides such as permethrin and etofenprox [3] may record higher mortality than cone tests at low concentrations of deltamethrin [9], presumably because of the longer potential exposure time, and care should be taken in interpreting the results of tunnel tests when evaluating non-irritant pyrethroids.

Nevertheless, a method for regeneration time was established, where the time required (in days) to reach a stable mortality level is the period required for regeneration of the net within a maximum of 7 days for practicality (if an ITN takes 7 days to regenerate, a laboratory test or a semi-field test of 20 washes takes 147 days instead of 20 days with a one-day interval). This is based on the idea that the net is now in “stable equilibrium” of insecticide within the net and on the surface so that no more insecticide will migrate to the surface. However, because the measurement relies on mosquito mortality it is bounded by the threshold to induce mortality among the mosquito strain tested. Therefore, the re-generation of enough bioavailable insecticide to kill 100% of pyrethroid susceptible mosquitoes may be reached in one day, and, as the net continues to regenerate, surface available insecticide sufficient to kill pyrethroid resistant strains may be reached in after a longer period [30]. Therefore, regeneration studies with fully susceptible mosquitoes on a new net where insecticide dosage is maximal, will always produce regeneration times much faster than the chemical equilibrium of the surface dosage. The regeneration time obtained in WHO bioefficacy tests is thus a mosquito strain dependent parameter and not a product parameter.

It was seen that a PE net that according to the standard method had a regeneration time of 1 day, in reality had around 5–7 days and that this prolonged as the dosage declined over a washing series [30]. This is in accordance with Fick’s second law of migration of an additive from a matrix to the surface. Modified to surface of a yarn, it can be described [31].

$$\frac{dC}{{dt}} = \frac{1}{r}\frac{d}{dr}\left( {rD\frac{dC}{{dr}}} \right).$$

It follows from this equation that the speed of migration of insecticide to the surface (*dC/dt*) depends on the concentration of the insecticide in the matrix, C, the yarn diameter, and the diffusion coefficient D. When the concentration declines over time due to repeated washes, so does the migration speed and therefore the regeneration time increases. Diffusion coefficient depends on the molecule and the polymer. In accordance with the free hole theory and the theory of diffusion in the amorph zones only, the diffusion coefficient can be correlated to the fraction of crystal zones to amorph zones. Reorganizing the crystal zones by a heat curing process (similar to [23]) provides a higher migration rate showing that this parameter is not just determined by the ratios of HDPE and LDPE with optimized molecular structures, but also by post extrusion processes including yarn stretching and heat curing [18].

### Why regeneration time matters

**Fig. 5:**
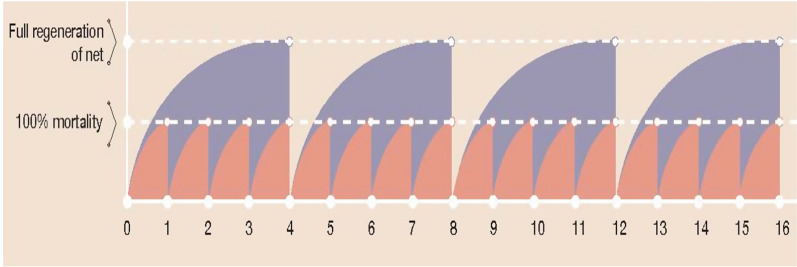
The impact of regeneration time on estimation of ITN wash resistance. The graphic describes what happens when a net is washed with shorter time interval (orange) than the real regeneration time (blue). Time to maximum regeneration (plateau of mosquito mortality) is 1 day whereas time to real regeneration (maximum bioavailable surface concentration of active ingredient (AI) is 4 days. On the Y axis we have an expression for amount of insecticide on the yarn surface. The 16 washes with 1-day interval removes approximately the same as 4 washes at the right interval

The importance of getting the regeneration time right is illustrated in Fig. 5. If a wash interval shorter than the time required for the net to regenerate is used then the net may fail in the assay after few washes because insufficient time is given for regeneration before the bioassay. Conversely, it may resist more washes since the maximum of insecticide available in the fibre or coating has not yet migrated to the surface resulting in a low amount of total insecticide removed per wash. The low curve in Fig. 5 shows a limited wash off at each washing with short intervals so many more washings can be withstood before ITN exhaustion than at the longer interval. In practice, it is advised that ITNs are washed no more often than every three months [32]. The nets thus have plenty of time to regenerate and to provide a lot more surface insecticide that will enhance efficacy but will be available for wash off or loss through evaporation. At the yarn surface, the total insecticide available is what was left at the surface at the production plus what has migrated from the coating or polyethylene matrix, minus what has evaporated and what was washed off. This is further complicated for the pyrethroids that are crystalline at room temperature, where there is an exchange between insecticide bound in crystal form and that in amorph molecular form [21]. The amorph has a much higher evaporation rate and a higher availability to the mosquito than the crystal form [22]. Crystals form slowly on the surface of ITNs and the larger crystals may have a lower surface area available to mosquitoes landing on the net, i.e., lower bioavailability [33].

### Surface concentration and chemical testing

Only insecticide at the surface of the yarns has any effect on mosquitoes. Measuring the total content of insecticide of a net sample is not a measure of bioavailability, although it can indicate the amount of insecticide lost through time due to washing, abrasion and evaporation. It is even possible to bind AI in the yarn that is not bioavailable. Polyester nets coloured after extrusion retain some insecticide that is not bioavailable [13] and there are patents in the literature that use pigment to bind Piperonyl Butoxide (PBO) in matrices. It is therefore important to use caution when considering chemical analysis of total insecticide content as a proxy for bioavailability during ITN durability monitoring or quality control.

### Implications of surface concentration for ITN evaluation and quality control

For ITNs designed to kill resistant mosquitoes it is important to establish the regeneration time for the target mosquito resistance profile, rather than assuming the regeneration time for a susceptible strain is adequate. Second, it is important to bioassay nets after an additional regeneration time interval post-washing to ensure reproducible results. Further, conduct of semi-field experimental tests of nets may be best started at least a week after washing is completed to better understand the true product performance under user conditions, where nets have time to fully regenerate between washes. This is not explicitly specified in existing guidance and may account for some of the heterogeneity seen between studies of the same product.

Monitoring ITNs longitudinally to measure their continued effectiveness under user conditions is therefore an essential additional step in ensuring ITN longevity after PQ Listing [34]. The additional step of comparing product performance (i.e., chemical content and bioefficacy) at 1, 2 and 3 years post-distribution against that of unwashed and 20 times washed products is a useful step for understanding product performance characteristics.

For quality control purposes, prior to programmatic distribution, ITN are evaluated for quality by measuring their chemical content. This does not adequately describe surface concentration. At the moment, the only recognized means of estimating surface concentration of insecticides is through WHO bioefficacy tests. However, as already mentioned this is a mosquito strain dependent parameter and not a product parameter. Methods to quantify surface concentration of insecticide and its relationship to product performance is urgently needed for standardization of product tests and for quality control. The cyanopyrethroid field test for deltamethrin nets [35] has been successfully linked with bioassay performance [36]. A method has been developed using net titration before and after washes in soap water or insolvents, that is designed to measure the time it takes for the surface concentration of one or more insecticides to become stable, i.e., the regeneration time (Skovmand, pers.commun.). Surface concentration of permethrin-treated nets has been assessed by rubbing the surface of an ITN with a cotton sample using a Martindale machine (normally used to determine the abrasion and pilling resistance of all kinds of textiles) and analysing the chemical on the cotton sample by HPLC, which correlated with bio efficacy via median time to knock down [37]. Greater uptake of existing methods is needed to generate the data required for recommendation of such tests as a regular part of product testing.

### Wash resistance index and wash resistance

Evaluating a net over 20 washes is too time consuming for regular quality control purposes. Therefore, the WHO devised wash resistance index (WRI) as part of ITN product specifications although the method is not linked to the regeneration time used for ITN evaluation [2]. It is not run at the same temperature for intervals between washes (40 °C for WRI versus 30 °C for the 20-wash process). The net is washed 4 times with one day interval and then the total insecticide content is determined. This method is problematic, because it includes insecticide at the surface before the very first wash. This surface insecticide is influenced by insecticide migration properties but also production method and storage. Especially in polyester nets, a large part of the insecticide is exposed to bioassay and washed off in the first wash. This means that log linear loss rate of insecticide assumed by the WRI is incorrect.

This can be seen by comparing total a.i. (g/kg) content of net after the first wash to that after 4 washes as in the WRI test, or from analysis after 1, 3 and 5 washes from the wash resistance test over 20 washes extracted from WHOPES reports [24, 38, 39] (Table 1). As wash resistance according to WHOPES [2] is not done at 4 washes, the mean of the values of 3 and 5 washes was used to compare to the WRI. For polyester nets, the highest percentage of wash off comes from the first wash, and the succeeding washes a smaller proportion is lost. The WRI test is often not predicting the average wash resistance of a net over 25 washes, but depends more on the very first wash off (Table 1). For polyethylene nets, less of the total insecticide is at the surface at the start, indeed the published data show often a negative wash off for the first wash, a result of sample variation bigger than wash off with one day interval. Finally, it can be seen that average wash off over 25 washes are closer between PE and PET nets than first wash off. By excluding the first wash off and taking the average wash off from washes 1 to 4 the WRI 1–4 for both PE and PET nets more closely approximates the average measured over 25 washes (Table 1).

**Table 1:** Considerations for calculating the wash resistance index

Polymer	Net/washes	0	1	3	“4”	5	25	WRI PS^c^	WRI 25^b^	WRI 4^a^	% Difference between WRI 4 and WRI 25	WRI 4 excluding the first wash (WRI1-4)^d^	% Difference between WRI 1–4 and WRI 25
PET	Safe Net pyr g/kg	7.50	6.32	6.08	5.65	5.22	3.46	92.5–97.0	97.2	93.2	3.75	97.24	0.28
	Washed off % per wash		15.7%	1.9%	7.1%	7.6%	1.7%						
PET	Interceptor pyr g/kg	4.44	4.20	3.55	3.36	3.17	0.65	90–101	92.6	93.3	0.90	94.57	0.40
	Washed off % per wash		5.5%	7.7%	5.4%	5.7%	4.0%						
PET	Interceptor G2 pyr g/kg	2.47	2.16	1.96	1.95	1.93	1.05	90–101	96.9	94.3	2.37	97.48	0.84
	Washed off % per wash		12.6%	4.6%	0.8%	0.8%	2.3%						
PE	Panda Net pyr pg/kg	1.83	1.84	1.81	1.81	1.81	1.65	95–101	99.5	99.7	0.14	99.59	0.00
	Washed off % per wash		− 0.5%	0.8%	0.0%	0.0%	0.4%						
PE	Miranet pyr g/kg	5.10	5.14	5.10	5.09	5.07	4.92	95–101	99.8	100	0.09	99.76	0.10
	Washed off % per wash		− 0.8%	0.4%	0.3%	0.3%	0.1%						
PE	Dawa 3 Roof pyr g/kg	2.40	2.45	2.25	2.05	1.85	1.02	90–100	99.8	96.1	0.50	95.64	0.99
	Washed off % per wash		− 2.1%	4.1%	8.9%	9.8%	2.2%						
PE	Dawa 4 pyr g/kg	2.44	2.43	2.44	2.42	2.39	2.37	90–100	99.8	99.8	0.09	99.9	0.01
	Washed off % per wash		0.4%	− 0.2%	1.0%	1.0%	0.0%						
PE	Veeralin pyr g/kg	7.38	7.29	7.19	7.23	7.27	6.85	95–100	99.8	99.5	0.21	99.5	0.03
	Washed off % per wash		1.2%	0.7%	− 0.6%	− 0.6%	0.3%						

Shows dosage of pyrethroids before wash and after 1, 3, 5, and 25 washes. Dosage after 4 washes is calculated as the mean between doses after 3 and 5 washes to get the value that could have been found in a classic WHO Wash Resistance Index test. Data extracted from [24, 38, 39]

^a^WRI calculated from the 4th root of the ratio of Active Ingredient at wash 4 to wash 0 as per CIPAC 4824 (WRI 4)

^b^WRI calculated from 25 washes (WRI 25)

^c^WRI stated in product specifications (WRI PS)

^d^WRI calculated after discarding the first wash:4th root of the ratio of Active Ingredient at wash 4 to wash 1 (WRI 1–4)

### Implications of WRI for ITN evaluation and quality control

If WRI was replaced by 1-WRI, thus measuring the wash off per wash rather than the amount of insecticide retained per wash, it is seen that the difference between WRI 97 and 99 is that 3 times more insecticide is washed off per wash, a significantly greater amount! For a net with a WRI of 80 to 100 means that 20% of total insecticide can be washed off per wash so very little is left after just 5 washes (just 15 months assuming the recommended user wash interval of every 3 months). It is, therefore, important for nets to be loaded with enough insecticide to withstand this wash off, or to have a WRI high enough that nets remain effective up to 20 washes.

The test method should therefore be changed in several ways. It is proposed to align the storage temperature between washes to the one used for the wash and bioassay test, 30 °C. Coatings and especially polyethylene are thermoplastic so the migration is temperature dependent, but not in a linear way. This will, however, increase the WRI value closer to 1 and make variations increase. This is compensated by replacing the 1-day interval with the recommended regeneration time. Finally, the very first wash off should be measured separately as it does not inform the wash resistance of the remaining insecticide. It is more accurate and meaningful to calculate the wash resistance on the fourth root of the ratio of insecticide remaining after wash 1 and wash 4 rather than the ratio of wash 0 and wash 4 (WRI 1–4 in Table 1).

### Sampling for tests

**Fig. 6:**
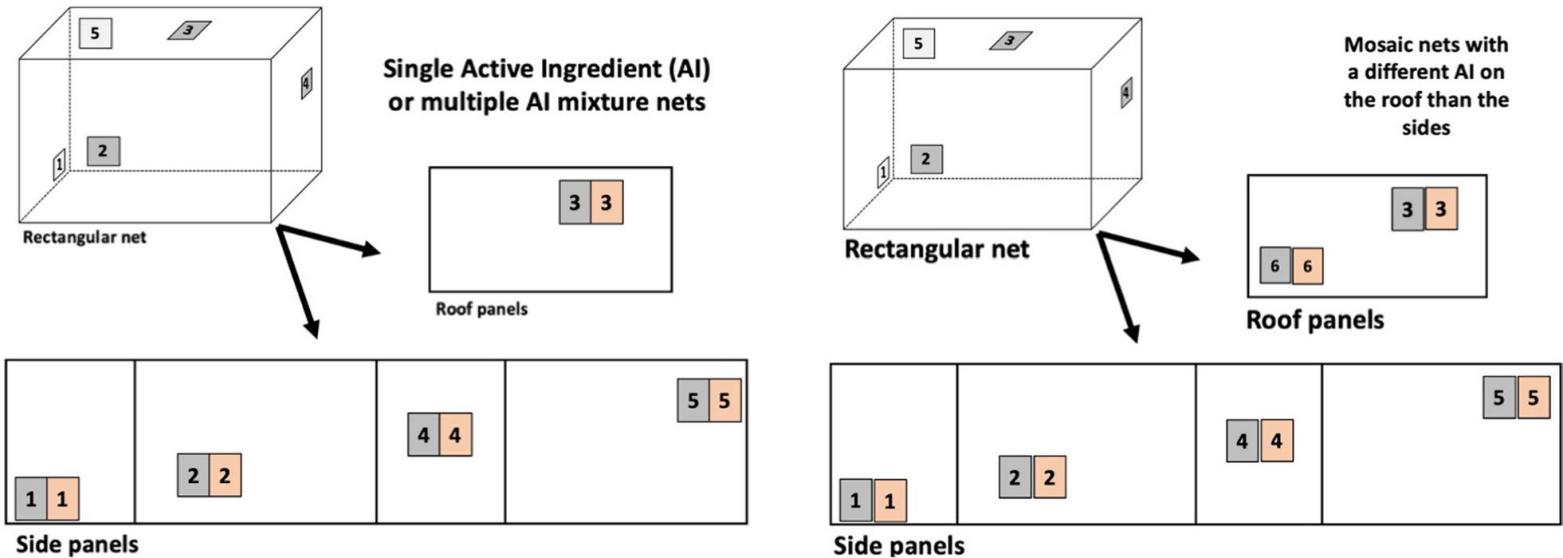
ITN sampling scheme. A Sampling scheme for 14 or 16 pieces of netting from each net, including positions HP1–HP5 (or HP6) for chemical assay. For bioassays of single active ingredient (AI) nets or mixture nets with the same AI on the roof and the sides, just 1 per side and roof are collected. For mosaic nets with different AI on the roof and sides two samples are collected from the roof

Bioassays and chemical testing of ITNs is conducted on swatches that are cut from the roof and the sides of the net as shown in Fig. 6. Since ITN technologies provide dosages with variation, it is important that net sampling for before and after studies e.g. washing or storage studies are collected to have the same start values. Otherwise, a washed net sample may contain more insecticide than an unwashed net sample simply because the samples were different before washing. This is illustrated in a WHOPES evaluation [29], where dosage in 3 out of 7 tested nets were higher after washing than before washing. The report states that for the test method to be reliable, intra-net sample variation must be less than 5%. However, the variability among products specifications reports up to 20% variability between samples from the same ITN. Since such results are not reliable, it can be concluded that this between sample variation exceeded 5%. To know the variation between nets, the sampling method of WHO is adequate, but to evaluate time series or wash series before and after samples must be used that are almost identical at start.

To illustrate this, 3 bed nets were sampled according to the WHO subsample method and each sample was analysed separately. The mean dosage per net varied from 2.54 to 2.60 g deltamethrin/net, but the coefficient of variation was from 7.0 to 10.0% per net. When 6 samples were taken from a 180 cm wide net in a way that each sample corresponded to the set of two bobbins on the knitting machine (band of 30 cm), the mean value was 2.54 g DM/kg net and the coefficient of variation was 12.8% between bands. Using samples across nets or between panels thus provides higher sample variation, higher than the expected wash off that typically is 1–5%.

When 3 bands were sampled along their length with 50 cm between 3 samples per panel, one from one end, one from the middle and one in the other end, the analysis showed that the 3 subsamples per band had coefficient of variation was 0.64, 0.84 and 1.45%. The average 0.99% is close to the coefficient of variation for the analytical method. Thus, there is only small dosage variations in a panel of a bed net along a single band and using several samples from the same band gives more reliable data for pre/post washing analysis. Measuring across panels, the inter-panel variation may result in negative values for wash off and other unreliable data because of the relatively high (> 1%) dosage variations between panels. Therefore, care should be taken to match chemical and bioassay data to samples cut along the same band.

When it comes from evaluating used nets in durability monitoring, data show that the roof of the nets is less damaged and generally retains more insecticide than the sides, probably because the sides are touched a lot more. Video observations of mosquitoes show that Afrotropical *Anopheles* mosquitoes spend 75% of their searching time on the roof part of the net [40], and a similar observation has been made for the central American *Anopheles albimanus* [41]. Studies have shown that even if the sides have no insecticide at all, the nets work perfectly well if the roof of the net is intact [42]. However, following the WHO sampling method, the roof only provides 25% of data used for generation of chemical analysis or bioassay results (1 out of 4 pieces). The 25% tolerance for insecticide content means a roof panel may be out of range or even untreated and the ITN may still pass quality assurance testing. The roof and sides of ITNs are produced from different runs or batches during production, and then stitched together. Considering the biological relevance of the roof, coupled with the likelihood of intra-net heterogeneity from different production runs, it is advisable to sample two swatches from the roof of any type of net when conducting durability monitoring or quality control. This is already done for mosaic nets that have different AI on the roof and sides (Fig. 6).

## Conclusions and recommendations

The WHO test methods rest on several assumptions that are not met. ITNs are not homogenous products. They very significantly within panels and between the sides and the roof. Therefore, a number of modifications to chemical assays and bioassays could improve test reproducibility.

Running tests of wash resistance using a before/after test on the same sample or band within a net will substantially reduce test variability so that the loss of insecticide from the fibres of the net with each wash can be more accurately estimated.

As mosquitoes frequently interact with ITN roofs and side and net panels are made from different production runs that can vary, additional sampling of the roof when evaluating ITNs is advisable. In nets where roof and sides are of the same material, the contribution of roof sample to the average (25% in durability monitoring or 20% for phase laboratory tests) is equal to or close to the tolerance for the specification (25%).

Mosquito mortality data cannot be reliably used for evaluating net surface concentration to determine regeneration time (RT) and resistance to washing as nets may regenerate beyond the insecticide concentrations needed to kill 100% of susceptible mosquitoes. Evaluation of regeneration time is clearly bounded by mosquito susceptibility to insecticides. Therefore, calculation of regeneration time of products designed for resistant mosquitoes using an appropriate resistant strain is a clear requirement. Thorough characterization of resistant strains before each evaluation, or close to the time of an evaluation is recommended since even standard strains vary in their phenotypic expression of resistance through time [43]. The use of resistant strains for evaluation of regeneration time of pyrethroid only nets may give supplementary information on true regeneration time, although the use of the median time to knock down [37] or median time to take off [30] may give a more sensitive measure of surface concentration using susceptible test systems. New videography methods that quantify mosquito interaction with the net surface show great promise in standardization of these measurements [44].

While bioassays are the best method currently available to test surface concentration, chemical assays to quantify surface concentration are urgently needed. Surface concentrations measurements to define product characteristics, such as WRI will both aid the development of more robust test methods for durability monitoring, and make linking of product specifications to product bioefficacy more realistic [45]. A number of methods are available in various stages of development. Using these methods in routine evaluations is not yet done and more work that incorporates both estimations of surface concentration and biological efficacy is required.

Consideration of the regeneration time (RT) is critical. While RT tends to be limited to seven days for practical reasons it is likely that incorporated nets will continue to regenerate for time periods beyond 7 days. The WHO guidelines do state that regeneration times beyond seven days may be used [2]. Test facilities should take care to allow products to fully regenerate before final bioassays at 20 washes, and before conducting experimental hut evaluations for optimal estimations of product performance.

The Wash Resistance Index (WRI) averaged over the first four washes is only informative if the product has a linear loss rate of insecticide, but it is highly influenced by the initial surface concentration. Using a wash resistance index that excludes the first wash off and uses the true regeneration time of the net gives more reliable results.

Storage conditions used for product specifications are lower than those encountered under product shipping and storage, that may exceed 50 ºC. This should be reconsidered as insecticide rate in ITN yarn and consequently ITN surface concentration and rate of loss of insecticide is highly temperature dependent.

Operational monitoring of new ITNs and linking observed product performance, such as bioefficacy after 2 or 3 years of use with product characteristics, such as WRI will aid the development of more robust test methods in the future and will help guide development of product specifications for new products coming to market.

## Data Availability

Data is available in WHOPES reports or can be obtained from Dr. Skovmand at reasonable request.
